# Gait Quality Assessment in Survivors from Severe Traumatic Brain Injury: An Instrumented Approach Based on Inertial Sensors

**DOI:** 10.3390/s19235315

**Published:** 2019-12-03

**Authors:** Valeria Belluscio, Elena Bergamini, Marco Tramontano, Amaranta Orejel Bustos, Giulia Allevi, Rita Formisano, Giuseppe Vannozzi, Maria Gabriella Buzzi

**Affiliations:** 1 Department of Movement, Human and Health Sciences, University of Rome “Foro Italico”, Interuniversity Centre of Bioengineering of the Human Neuromusculoskeletal System, P.zza Lauro de Bosis 15, 00135 Roma, Italy; v.belluscio@studenti.uniroma4.it (V.B.); elena.bergamini@uniroma4.it (E.B.); m.tramontano@hsantalucia.it (M.T.); a.orejelbustos@studenti.uniroma4.it (A.O.B.); 2 IRCSS Fondazione Santa Lucia, Via Ardeatina 306, 00179 Roma, Italy; giulia.allevi@gmail.com (G.A.); r.formisano@hsantalucia.it (R.F.); mg.buzzi@hsantalucia.it (M.G.B.)

**Keywords:** wearables, inertial sensors, traumatic brain injury, dynamic balance, gait disorders, gait patterns, head injury, gait symmetry, gait smoothness, acceleration

## Abstract

Despite existing evidence that gait disorders are a common consequence of severe traumatic brain injury (sTBI), the literature describing gait instability in sTBI survivors is scant. Thus, the present study aims at quantifying gait patterns in sTBI through wearable inertial sensors and investigating the association of sensor-based gait quality indices with the scores of commonly administered clinical scales. Twenty healthy adults (control group, CG) and 20 people who suffered from a sTBI were recruited. The Berg balance scale, community balance and mobility scale, and dynamic gait index (DGI) were administered to sTBI participants, who were further divided into two subgroups, severe and very severe, according to their score in the DGI. Participants performed the 10 m walk, the Figure-of-8 walk, and the Fukuda stepping tests, while wearing five inertial sensors. Significant differences were found among the three groups, discriminating not only between CG and sTBI, but also for walking ability levels. Several indices displayed a significant correlation with clinical scales scores, especially in the 10 m walking and Figure-of-8 walk tests. Results show that the use of wearable sensors allows the obtainment of quantitative information about a patient’s gait disorders and discrimination between different levels of walking abilities, supporting the rehabilitative staff in designing tailored therapeutic interventions.

## 1. Introduction

Head injuries are considered a major health problem as they are associated with high mortality and disability in young adults (<45 years of age) [1,2]. Nearly 70% of all brain-injury cases are males [3], and most events are caused by falls (28%), followed by motor vehicle accidents (20%) and blows (19%) [4]. Traumatic brain injuries (TBI) impress a significant burden on the health care system, due to the need for therapy to address physical, communicative, and psychological problems [5]. Costs are usually more elevated when the traumatic brain injury is considered severe [5]; that is with an initial Glasgow coma scale score (GCS) of 8 or less [6]. Neuropsychological and cognitive impairments, such as anxiety and depression, selective/sustained attention, language, and executive function deficits have been well documented in the literature [7,8,9,10,11,12]. Less attention has been placed on motor impairments, in striking contrast with available data on other neurological populations, such as stroke and Parkinson’s disease ones [13,14,15,16,17,18]. The available studies mainly focused on impaired balance and altered coordination. Specifically, Rinne and colleagues [19] described that well-recovered men with TBI had impaired balance and agility compared to healthy controls. A recent review performed by Williams and colleagues [20] evidenced that people with TBI walked more slowly than healthy controls, primarily due to reduced step length. A few authors emphasized the impact of post-traumatic parkinsonism or post-traumatic cerebellar syndrome [21,22], two conditions that interfere with walking and balance performances in persons surviving from TBI. Additionally, balance abnormalities have also been reported in terms of increased postural sway during quiet standing or functional tasks, with altered sensory inputs [23,24,25]. Additionally, gait analysis has been used in few studies: Chou and colleagues [26] showed that people who suffered from TBI usually present a gait pattern with a significantly slower speed and a shorter stride length, confirming previous results [27]. Basford and colleagues [28] reported that gait analysis, balance, and vestibular testing could document subtle biomechanical changes among participants with TBI, suggesting the appropriateness of gait and balance testing in this population, even when motor disorders are not clinically evident. 

Taken together, evidence exists for persistent motor deficits after TBI. However, these studies have focused on mild (GCS > 13) and moderate (GCS between 9 and 13) TBI, and to the authors’ knowledge, no quantitative information is available about motor ability in people who have incurred a severe TBI (sTBI). An objective characterization of their level of motor impairment could be an important step in the rehabilitation process of this population, helping in obtaining not only physical improvements, but also increasing the independence in daily life and the overall quality of life. This characterization, in order to be helpful and informative, should be ecological and as non-intrusive as possible.

In this framework, attention is growing on miniaturized and wearable instruments that quantify movement patterns in a non invasive way: inertial measurement units (IMUs), embedding accelerometers and gyroscopes, have been widely used in the last two decades since they present many advantages compared to the traditional gait analysis approach based on stereophotogrammetry and force platforms. From the data measured by these units, spatiotemporal gait parameters [29] and stability-related parameters [13,30] can be extracted, allowing fall risk to be assessed [31], and allowing one to differentiate gait patterns between healthy and pathological populations [13,32,33,34]. However, in the sTBI population, an instrumented approach with IMUs has never been proposed and no information is available about their capability to discriminate among different levels of walking ability, as defined by currently administered clinical scales, such as the dynamic gait index scale [35]. An integrated approach based on the “gold standard” clinical evaluation method which relies on clinical scales and the proposed sensor-based assessment would overcome the limitations of a subjective evaluation, depending on the operator’s specific training, helping in revealing changes hardly detectable using clinical scales. In addition, this integration would allow to assess patients in ecological contexts, where they perform tasks more similarly to those of real life, providing objective motor ability characterization.

Given these premises, the aims of the present study were twofold: (i) to quantify gait patterns in sTBI population using a set of wearable inertial sensors; (ii) to investigate the association of the estimated gait quality indices with the level of walking ability and the scores of commonly administered clinical scales. Specifically, spatiotemporal parameters and gait quality indices (dynamic stability, symmetry, and smoothness) were investigated considering clinical performance tests commonly used in the routine assessment [36,37]. 

The hypothesis is that the instrumental approach could be a valid support to the traditional clinical evaluation in order to obtain quantitative and objective information about sTBI patients’ motor impairments, discriminating between different levels of walking abilities, and helping clinicians with defining and evaluating the efficacy of personalized rehabilitation treatments, as previously reported in the literature [38]. Furthermore, the correlation analysis could help with simplifying and facilitating routine evaluation in terms of time-consuming administration of clinical scales, possibly allowing a reduction in the number of scales used, maintaining those necessary to characterize the investigated population/motor task.

## 2. Materials and Methods

The research was performed at the Santa Lucia Foundation and it was approved by the Local Independent Ethics Committee of Fondazione Santa Lucia IRCCS (Rome, Italy) (protocol number: CE/PROG.700).

### 2.1. Participants

Twenty healthy subjects (control group, CG) (age: 33.9 ± 9.5 years), 15 males and 5 females, and 20 people who suffered from a sTBI (age: 33.4 ± 10.5 years), 15 males and 5 females, were involved in the study. This sample size complied with the minimum number of participants recommended by a power analysis purposely performed (α = 0.05; power (1-β) = 0.95, effect size d: 0.7) for non parametric comparisons [39]. Exclusion criteria for CG were the presence of any orthopedic, neurological, or other co-morbidities which could have influenced the motor performance. Inclusion criteria for sTBI were: (i) age between 15 and 65 years; (ii) Glasgow coma scale (GCS) score ≤ 8 (used to objectively describe the severity of impaired consciousness at the time of injury) [6]; (iii) level of cognitive functioning (LCF) ≥ 7 [40]; (iv) presence of disturbances in static and dynamic balance; (v) ability to understand verbal commands. Almost all the patients selected suffered from a sTBI as a consequence of a traffic accident (19 out 20 participants), whereas one person suffered from a sTBI due to a fall. 

### 2.2. Procedures

#### 2.2.1. Clinical Assessment

The following clinical scales were administered by an expert physiotherapist to all sTBI participants, to assess static and dynamic balance, ambulation skills, and mobility deficits:

- Dynamic gait index (DGI)—to assess a subject’s ability to modify gait in response to changing task demands. It consists of items rated from 0 to 3 (0 = severely impaired; 3 = normal performance), yielding a maximum score of 24 points. A score lower than 19 points has been associated with impairment of gait and fall risk [35,41].
- Berg balance scale (BBS)—to measure 14 different tasks related to balance and postural control. It is scored from 0 to 4, with 0 indicating that the subject is unable to perform the task and 4 that the subject fully meets the most difficult criteria required for the task [42].
- Community balance and mobility scale (CB&M)—to assess specific aspects of balance and mobility which are necessary for independent functioning within the community [43]. This scale includes several challenging tasks and it is based on 19 tests. Higher scores are indicative of better balance and mobility.

To codify for different levels of walking ability, sTBI patients were further divided into two sub-groups, according to their score in the dynamic gait index clinical scale: persons with a score >19 were considered severe (10 people, sTBI-1), while those with a score ≤ 19 were considered very severe (10 people, sTBI-2), according to [35]. The demographic characteristics of each subgroup are reported in Table 1.

#### 2.2.2. Motor Assessment

Each participant was asked to perform three different motor tasks in a randomized order: the 10 m walk Test (10mWT), the figure-of-8 walk test (F8WT), and the Fukuda stepping test (FST). All tests were carried out in a fully dedicated quiet area at the Santa Lucia Foundation, where the surface was accurately kept flat, and participants were asked to stay barefoot and to stand upright for at least 5 s at the beginning and at the end of each trial. Tasks were carefully explained and demonstrated by an instructor before the testing. The instructor also gave the patients start and stop commands and stayed close to participants to prevent dizziness and/or falls. A detailed description of the motor tasks is reported below.

10 m Walk Test (10mWT)

The 10mWT is a widely used and recommended test for measuring gait speed in different populations [44]. The experimental protocol of the assessment was selected according to previous studies [13,45]: it consists of walking on a straight 14 m long walkway for three repetitions at the participant’s preferred walking pace, with the middle 10 m marked on the floor and considered as steady-state walking for further analysis. The time taken to walk the middle 10 m was measured using a stopwatch and walking speed was calculated by dividing the distance covered (i.e., 10 m) by the time taken.

Figure-of-8 Walk Test (F8WT)

**Figure 1:**
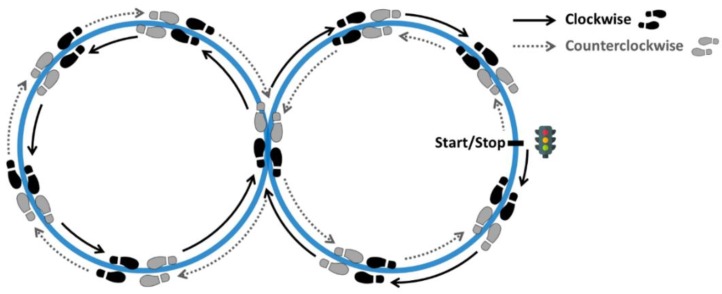
Figure-of-8 shape used for the figure-of-8 walk test (F8WT). Clockwise and counterclockwise directions are indicated with grey and black arrows, respectively.

The F8WT requires a person to walk a figure-of-8 shape, as illustrated in Figure 1, marked on the floor with tape, with each circle diameter of 1.66 m (5.44 ft) [46]. Participants were instructed: (*i*) to stand still with feet side-by-side in the start position facing the “8”; (*ii*) to begin walking at their preferred pace when ready; (*iii*) to stop when returning to the start position, placing feet side-by-side again. The test was performed three times for each F8WT direction (clockwise and counterclockwise), alternating the two directions, and the entire trial was considered for further investigations. 

Fukuda Stepping Test (FST)

The FST is a test used for the diagnosis of vertigo-associated disease [47] and an instrumented version of this test has been recently proposed in the literature [48] and was adopted in this work. Participants were instructed to stand upright blindfolded with both arms frontally outstretched, creating a 90° angle between the arms and the body. Then, they were asked to step on the spot for one minute and to remain still in the final position. Lateral and forward displacements, as well as the amount and side of rotation, were marked on the floor by a piece of tape and subsequently reported as clinical FST parameters. For what concerns the sensor-based parameters, the first and last three strides were discarded in order to evaluate only steady-state stepping. 

### 2.3. Equipment

While performing the three above mentioned motor tasks, each participant was equipped with five synchronized inertial measurement units (IMUs) (128Hz, Opal, APDM, Portland, Oregon, USA): one located on the occipital cranium bone close to the lambdoid suture of the head (H), one on the center of the sternum (S), and one at L4/L5 level, slightly above the pelvis (P), and were used to assess the upper-body stability. The other two IMUs were located on both shanks, slightly above the lateral malleoli, and were used for step and stride segmentation. Each IMU was securely fixed to the participant’s body with Velcro straps, except for the head IMU, which was inserted in a tailored pocket of a swim cap worn by each subject. 

### 2.4. Data Processing 

All data processing was performed using the Matlab software (The MathWorks Inc., Natick, MA, USA). Each unit embedded three-axial accelerometers and gyroscopes (±6 g with g = 9.81 m∙s^−2^, and ±1500 °/s of full-range scale, respectively) and provided the quantities with respect to a unit-embedded system of reference. To guarantee a repeatable reference system for the three IMUs located on the upper body, each unit was aligned with the corresponding anatomical axes (antero-posterior: AP, medio-lateral: ML, and cranio-caudal: CC) following the procedure proposed by [49]. The following spatiotemporal parameters were obtained, through a peak detection algorithm, on the ML angular velocity signals measured by the two IMUs on the shanks: average stride duration (SD = time to complete the test/total number of strides) and average stride frequency (SF = total number of strides/time to complete the test). The following gait quality indices were estimated:

- Normalized root mean square (nRMS) values of the accelerations were calculated by dividing the RMS, AP, and ML components by the CC component, at each upper-body level (P, S, H). High RMS values have been associated with higher amount of acceleration, and hence, decreased stability, as reported in [29].
- Attenuation coefficients (AC) [50] between each level pair of the upper-body, for each acceleration component (*j*), defined as:
  $$ACPS_{j}=\left(1-\frac{RMS_{j}S}{RMS_{j}P}\right),$$
  $$ACPH_{j}=\left(1-\frac{RMS_{j}H}{RMS_{j}P}\right),$$
  $$ACSH_{j}=\left(1-\frac{RMS_{j}H}{RMS_{j}S}\right).$$
  Each coefficient represents the variation of the acceleration from lower to upper-body levels. A positive coefficient indicates an attenuation of the accelerations, while a negative coefficient indicates an amplification of the accelerations from the lower to the upper body level.
- Improved harmonic ratio (iHR), as proposed by [51], was calculated for each acceleration component (*j*) measured at the pelvis level. This index is based on a spectral analysis of the acceleration signals and is a measure of hemilateral symmetry when stepping (0% = total asymmetry; 100% = total symmetry). It was calculated as follows:
  $$iHR_{j}=\frac{\sum Power\;of\;intrinsic\;harmonics}{\sum Power\;of\;intrinsic\;harmonics+\sum Power\;of\;extrinsic\;harmonics}\cdot 100.$$
- SPectral ARC length (SPARC), as proposed by [52], calculated for each acceleration component (*j*) measured at the pelvis level. The calculation of SPARC was performed as follows:
  $$-\int_{0}^{{ῶ}_{c}}[{\left(\frac{1}{{ῶ}_{c}}\right)}^{2}+{\left(\frac{dA\left(ῶ\right)}{dῶ}{)}^{2}\right]}^{\frac{1}{2}}dῶ;A\left(ῶ\right)=\frac{A\left(ῶ\right)}{A\left(0\right)}$$
  $${ῶ}_{c}=min\{{ῶ}_{c}{}^{max}min\{ῶ\vee A\left(r\right)\langle Á,\forall r\rangle ῶ,$$
  where A(ῶ) is the Fourier magnitude spectrum of the acceleration signal a(t) and A(ῶ) is the normalized magnitude spectrum.

### 2.5. Statistical Analysis

Descriptive and inferential statistical analyses were performed using IBM SPSS Statistics software (v23, IBM Corp., Armonk, NY, USA), and the alpha level of significance was set at 0.05. The normal distribution of each parameter was verified using the Shapiro–Wilk test. As most of the parameters were not normally distributed, the following non-parametric tests were performed:

- Mann–Whitney U test to investigate if significant differences existed between sTBI-1 and sTBI-2 for the clinical scale scores;
- Kruskal–Wallis H-test on the estimated biomechanical parameters, to investigate if significant differences existed among the different levels of walking ability (‘‘group” factor: CG, sTBI-1, or sTBI-2);
- Spearman’s rank correlation coefficient (q) between gait quality indices and clinical scale scores, considering the whole sTBI group.

## 3. Results

### 3.1. Clinical Scale Score Results

The scores of the administered clinical scales for sTBI-1 and sTBI-2 are reported in Table 2. Results show that sTBI-2 group (defined as very severe TBI according to DGI scores; see methods) presented worse, statistically significant scores in the three clinical scales compared to sTBI-1.

### 3.2. Spatio-Temporal Parameters and Clinical FST Parameters 

Results of temporal (stride frequency and stride duration) and clinical FST parameters (lateral and forward displacements; amount and side of rotation) for the three groups are reported in Table 3. Statistically significant differences were present for all three motor tasks when comparing CG with sTBI-2 and sTBI-1 with sTBI-2. In addition, statistically significant differences between CG and sTBI-1 were found in the spatio-temporal parameters of the FST. Concerning clinical FST parameters, no statistical differences are displayed in terms of lateral and forward displacements, or amount and side of rotation among the three groups. Walking speeds (mean ± standard deviation) obtained during the 10mWT were: 1.48 ± 0.20, 1.10 ± 0.23, and 0.53 ± 0.20, for the CG, sTBI-1, and sTBI-2 groups, respectively. Significant differences were found between CG and both sTBI-1 and sTBI-2, as well as between sTBI-1 and sTBI-2. 

### 3.3. Root Mean Square, Attenuation Coefficients, Improved Harmonic Ratio, and SPARC

**Figure 2:**
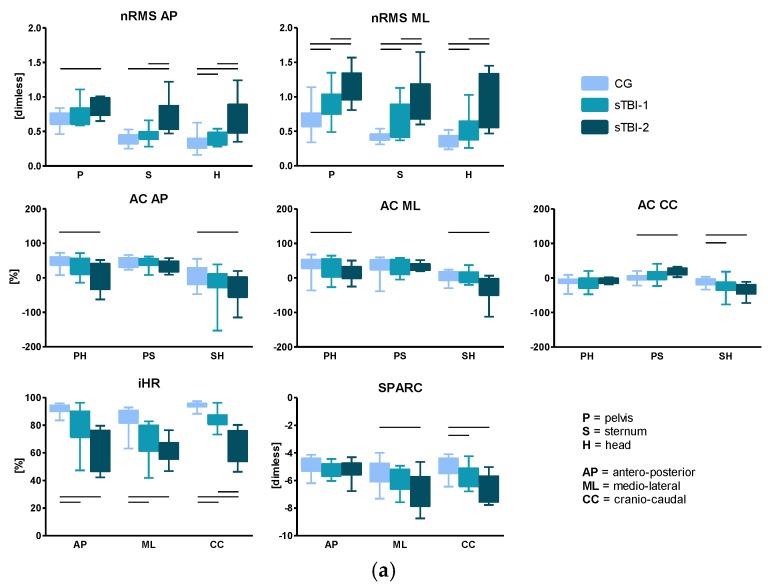

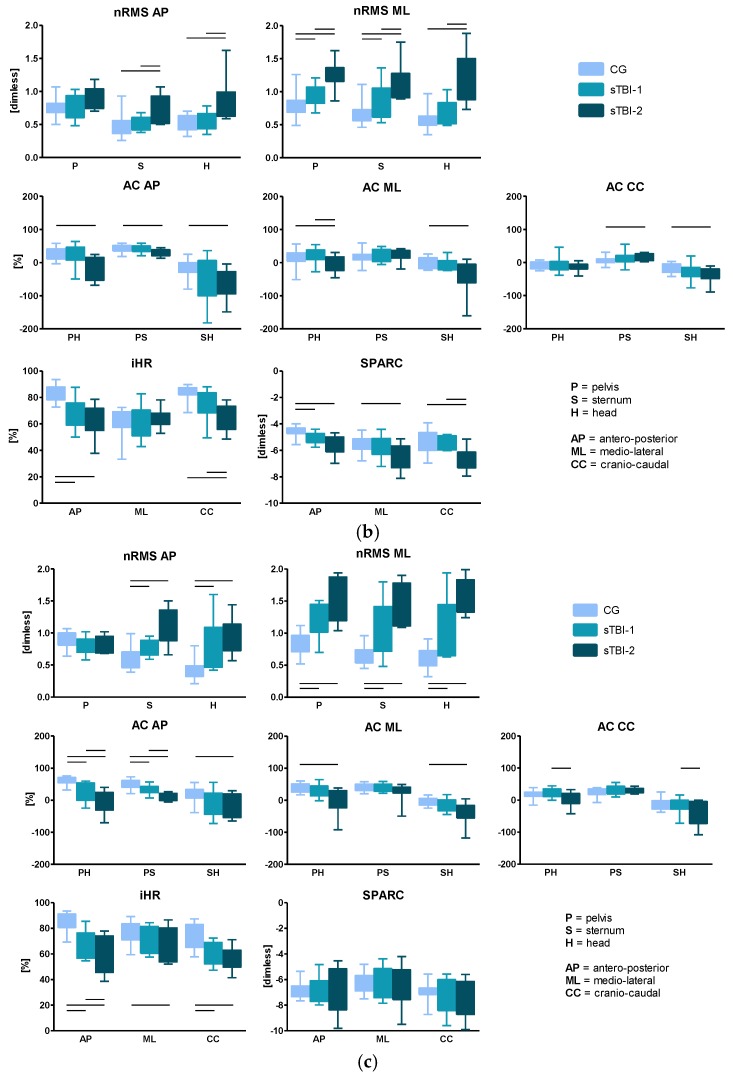
Normalized root mean square (nRMS) values, attenuation coefficients (AC), improved harmonic ratio (iHR), and SPectral ARC length (SPARC) for the sTBI sub-groups and for CG in 10mWT (**a**), F8WT (**b**), and FST (**c**). Medians and interquartile ranges are reported. AP, antero-posterior; ML, medio-lateral; CC, cranio-caudal; P, pelvis; S, sternum; H, head. The horizontal lines indicate statistically significant between-groups differences. (**a**) 10 m walk test. (**b**) Figure-of-8 walk test. (**c**) Fukuda stepping test.

Significant differences were found for the three motor tasks (10mWT, F8WT, and FST) when comparing both sTBI against CG and sTBI-1 against sTBI-2. Results regarding the 10mWT, the F8WT, and the FST are reported in Figure 2a–c, respectively. 

### 3.4. Association of the Gait Quality Indices with the Clinical Scale Scores

Correlation analysis (Table 4) shows that several indices displayed a significant correlation with the clinical scales scores in the three motor tasks, especially in the 10mWT and F8WT. 

## 4. Discussion

The aims of this study were to quantify gait quality of a sTBI population with different levels of walking ability using a set of wearable inertial sensors and to investigate the association of the estimated gait quality indices with the scores of commonly administered clinical scales. Results show that the instrumented approach allows (*i*) obtainment of quantitative and objective information about patient’s motor impairments; (*ii*) discrimination between different levels of walking abilities; (*iii*) exploration of the relationship between the estimated gait quality indices and the clinical scale scores. As expected, clinical scale scores displayed a consistent increasing trend from low to high walking ability levels, showing statistically significant differences between severe (sTBI-1) and very severe (sTBI-2) TBI participants (Table 2). A similar trend was observed when considering the spatio-temporal parameters: for what concerns walking speed, statistically significant differences were found between the control group (CG) and sTBI-2, and between sTBI-1 and sTBI-2 (Table 3). These results are consistent with the existing literature about healthy people [13,53] and TBI participants [26], and confirm the relevance of walking speed as an informative and concise parameter to discriminate between different level of walking ability. 

In addition, the values of stride frequency and stride duration obtained in this study are consistent with previously reported results. In particular, in persons with sTBI, a reduced stride frequency, along with an increased stride duration, may be related to post-traumatic Parkinsonism [22,54,55]. Furthermore, as suggested in [56], it can be speculated that people with sTBI increase their stride duration in order to compensate for gait instability and counteract the fear of falling. This significant gait impairment was still observed despite the provision of optimal medication therapy, confirming the very close relationship between altered gait and postural instability in this population [57,58]. 

Interesting results come from the estimated gait quality indices: almost all parameters in the three motor tasks were able to discriminate between CG and both sTBI groups, especially the sTBI-2, as expected. The actual added value of the proposed approach, however, lies in its ability to detect possible differences between sTBI-1 and sTBI-2, facilitating discriminating between different levels of walking ability. In this respect, in the 10mWT, the two sTBI sub-groups presented differences in gait stability and symmetry. Specifically, considering gait stability, sTBI-2 showed higher nRMS compared to sTBI-1. High nRMS values have been associated with a higher amount of acceleration, and hence, decreased stability [13,29,30,33,34,50]. Both sTBI subgroups, and especially sTBI-2, displayed a decreased stability at the three upper body levels, particularly in the ML direction. This is consistent with previous studies dealing with other neurological populations [13,15,59]. In addition, the attenuation coefficient from pelvis to head in the ML direction discriminates between the two sTBI sub-groups, highlighting that the sTBI-2 sub-group exhibits a limited bottom-up attenuation of upper body accelerations. This result is related to a lack of ability to stabilize the head, impairing the consequent planning of adaptive motor strategies. Concerning gait symmetry, the iHR in the CC component discriminated between sTBI-1 and sTBI-2, showing a reduced gait symmetry, particularly evident in the sTBI-2. Reduced symmetry has been widely associated with an increased fall risk [60,61], thus indicating this parameter as a biomarker for the identification of patients at high risk of falling. 

In the F8WT, the nRMS and iHR discriminated between sTBI-1 and sTBI-2, as reported for the 10mWT. In addition, differences were also pointed out considering the smoothness: in fact, the SPARC discriminated sTBI-1 and sTBI-2 well, probably because of greater upper body rigidity in sTBI-2 than sTBI-1 observed in this more difficult task. It is worth mentioning that, being characterized by a curved trajectory, the execution of the F8WT involves the activation of different cortical areas than those required in the planning of straight point-to-point movements. In fact, it is well known that the trajectory planning during curved-path conditions requires additional preparation time [62,63]. The results of the present study show indeed that the F8WT seems to be the walking test that better discriminates among different walking ability levels. This suggests that testing dynamic balance abilities during curved trajectories could be useful for assessing gait in conditions more relevant to cognitive-motor dual tasks, and thus, closer to daily living activities [64,65]. 

For what concerns the FST, results about clinical FST parameters confirm the previous literature [48,66]: no differences were found in terms of AP–ML displacements, nor side and degree of rotation among groups. The presence of rotation in either direction in the CG shows that turning while stepping on the spot also occurs in healthy people, confirming that clinical FST parameters are not able to distinguish between healthy and pathological subjects, confirming the doubts about its clinical use also for this population. Conversely, when considering gait quality indices obtained from the instrumented FST, the discrimination capability of the test greatly increased: in fact, significant differences were found, not only between CG and both sTBI sub-groups, but also between the sTBI sub-groups. Specifically, for what concerns stability, the nRMS did not discriminate between different levels of walking abilities in pathological subjects, as observed in the 10mWT and the F8WT. On the other hand, attenuation coefficients in the AP and CC directions distinguished between sTBI sub-groups, with sTBI-2 showing less ability in attenuating upper body accelerations from lower to higher levels. Additionally, a reduced symmetry was displayed by sTBI-2 with respect to sTBI-1, with the AP component of the iHR displaying a significant difference between the two sub-groups. The AP direction seems to be the most critical in very severe TBI and this result is in agreement with the existing literature about stroke patients [48], indicating the AP component as the most informative when comparing patients with different walking abilities. In addition, the absence of the visual input during the FST plays an important role on the sensory reweighting, which has been acknowledged as critical in the TBI population [67]. Therefore, these results confirm that the instrumented approach in this test provides valuable information about patients’ motor strategies and useful data to tailor rehabilitation protocols [48].

When considering the second aim of the study, several correlations were found between clinical scales and gait quality indices, especially in the 10mWT and the F8WT. nRMS and attenuation coefficients, parameters related to dynamic stability, correlated well with all clinical scales, while worse correlations were present when considering the iHR and the SPARC in both the 10mWT and the F8WT, with no correlations at all for these two parameters in the FST. It should be acknowledged that the proposed clinical scales do not consider tests in which the visual input is removed: this could be one of the reasons why only few correlations have been found when considering the FST. These results highlight the lack of specificity that some clinical scales exhibit [68], while confirming their ability to determine whether or not a patient has a motor impairments. Therefore, the integration of traditional scales and technology-based protocols could assist with improving current clinical routines and with designing rehabilitation treatments, helping to bringing more sensitive, specific, and responsive motor tasks to clinical practice. 

Despite the promising results, this study presents some limitations: the main limitation is the heterogeneity of the sample, mainly due to the severities and the locations of the brain injuries. Increasing the sample would likely lead to reduce the heterogeneity of the sample. Furthermore, the relationship between gait characteristics and specific neurological deficits, such as post-traumatic parkinsonism or cerebellar syndromes, and the presence of possible cognitive and behavioral sequelae of sTBI, were not investigated. Although such analyses were beyond the scope of the present study, they could be considered in further studies, in order to obtain more detailed information and better discriminate among people suffering from sTBI. 

## 5. Conclusions

People who suffer a sTBI often complain of balance and gait impairments, but despite the evidence that neuromotor deficits are a common consequence of a sTBI, the existing literature does not adequately describe balance strategies adopted by sTBI survivors. This lack of information depends on various factors: the heterogeneity and severity of the brain damage, the patient’s age, and the presence of pre-morbid/co-morbid conditions are the most significant. Furthermore, subtle cognitive functioning deficits, such as executive functions, which are detectable even in persons with good recovery after sTBI [7,63], may interfere with dynamic performances. 

The main contribution of the present work is represented by the analysis of gait stability, symmetry, and smoothness indices which objectively describe gait quality in patients with sTBI. Specifically, the lack of ability of both severe and very severe TBI patients to stabilize their head by attenuating body accelerations may have a big impact. In fact, the vestibular system is located at head level; therefore, a high head acceleration could be critical for the planning of adaptive motor strategies. 

The data reported herein suggest the appropriateness of an integrated assessment using both clinical scales and wearable sensors to objectively evaluate gait and balance impairments during different dynamic tasks. This integrated approach may be useful to assessing the measures of changes during rehabilitation training aimed at improving patients’ gait quality and limiting the risk of falling, supporting rehabilitative staff with designing effective and tailored interventions.

## Figures and Tables

**Table 1:** Demographic and anthropometric characteristics of the control group (CG), severe traumatic brain injury 1 (sTBI-1), and sTBI-2. Mean ± standard deviation values are displayed.

	CG	sTBI-1	sTBI-2
Nr. of Participants	20	10	10
Nr. of Males	15	8	7
Age [Years]	33.9 ± 9.5	33.2 ± 9.6	36.1 ± 13.1
Body Mass [kg]	78.3 ± 14.9	75.9 ± 16.2	71.0 ± 14.7
Body Height [m]	1.78 ± 0.09	1.73 ± 0.11	1.70 ± 0.11
Time Since Trauma [days]	-	308 ± 182	512 ± 476

**Table 2:** Clinical scales results for sTBI. Mean ± standard deviation values are displayed. Statistically significant differences are indicated with *.

	sTBI-1	sTBI-2	*p*-Value
Dynamic gait index (DGI)	22.1 ± 1.7 *	15.0 ± 3.0 *	0.000
Berg balance scale (BBS)	49.8 ± 2.1 *	42.4 ± 3.9 *	0.000
Community balance and mobility scale (CB&M)	42.0 ± 14.0 *	15.5 ± 8.9 *	0.000

**Table 3:** Temporal and FST parameters. * indicates statistically significant differences between CG and sTBI-2 (*p* < 0.001); ^§^ indicates statistically significant differences between sTBI-1 and sTBI-2 (*p* < 0.05); ^#^ indicates statistically significant differences between CG and sTBI-1 (*p* < 0.001). Clinical FST parameters: the values of the antero-posterior (AP) and medio-lateral (ML) displacements, the amount of rotation and the side of rotation of the three groups of subjects (CG, sTBI-1, sTBI-2) in the three tasks are reported (mean ± standard deviation).

		Stride Frequency	Stride Duration	Rotation	Side	Displacement	
						AP	ML
		[Strides^x^s^−1^]	[s]	[Degrees]	[% Right]	[cm]	[cm]
**10mWT**	CG	0.9 ± 0.0 *	1.1 ± 0.1 *	-	-	-	-
	sTBI-1	0.8 ± 0.1 ^§^	1.2 ± 0.1 ^§^	-	-	-	-
	sTBI-2	0.7 ± 0.1 *^,§^	1.4 ± 0.2 *^,§^	-	-	-	-
**F8WT**	CG	0.8 ± 0.1 *	1.2 ± 0.1 *	-	-	-	-
	sTBI-1	0.8 ± 0.1 ^§^	1.2 ± 0.2 ^§^	-	-	-	-
	sTBI-2	0.7 ± 0.1 *^,§^	1.5 ± 0.2 *^,§^	-	-	-	-
**FST**	CG	0.8 ± 0.1 *^,#^	1.2 ± 0.2 ^#^	66 ± 66	30	146 ± 71	44 ± 33
	sTBI-1	0.6 ± 0.2 ^§,#^	1.8 ± 0.9 ^#^	27 ± 17	40	141 ± 38	45 ± 46
	sTBI-2	0.5 ± 0.2 *^,§^	2.0 ± 1.4	28 ± 23	50	101 ± 60	27 ± 31

**Table 4:** Spearman’s correlation coefficients (*p*) between each estimated parameter and each clinical scale. Statistical significance is indicated by asterisks (* *p* < 0.05; ** *p* < 0.001). Abbreviations: BBS, Berg balance scale; DGI, dynamic gait index; CB&M, community balance and mobility scale, RMS, root mean square; AC, attenuation coefficient; iHR, improved harmonic ratio; SPARC, spectral arc length; AP, antero-posterior; ML, medio-lateral; CC, craniocaudal; P, pelvis; S, sternum; H, head.

			10mWT			F8WT			FST	
		BBS	DGI	CB&M	BBS	DGI	CB&M	BBS	DGI	CB&M
**RMS_P**	AP	−0.243	−0.309	−0.254	−0.337	−0.335	−0.398	0.043	0.103	0.118
	ML	−0.656 **	−0.467 *	−0.605 **	−0.730 **	−0.666 **	−0.819 **	−0.404	−0.445	−0.500 *
**RMS_S**	AP	−0.555 *	−0.585 **	−0.679 **	−0.491 *	−0.484 *	−0.600 **	−0.495 *	−0.655 **	−0.500 *
	ML	−0.583 **	−0.503 *	−0.733 **	−0.571 *	−0.463 *	−0.749 **	−0.460 *	−0.516 *	−0.695 **
**RMS_H**	AP	−0.674 **	−0.641 **	−0.712 **	−0.594 **	−0.611 **	−0.665 **	−0.353	−0.309	−0.246
	ML	−0.781 **	−0.705 **	−0.821 **	−0.796 **	−0.708 **	−0.839 **	−0.618 **	−0.506 *	−0.660 **
**ACPH**	AP	0.535 *	0.577 **	0.451	0.481 *	0.349	0.418	0.608 **	0.550 *	0.512 *
	ML	0.493 *	0.491 *	0.595 **	0.631 **	0.598 **	0.637 **	0.630 **	0.481 *	0.623 **
	CC	−0.061	−0.076	0.004	0.057	0.059	0.182	0.544 *	0.551 *	0.567 *
**ACPS**	AP	0.495 *	0.443	0.588 **	0.477 *	0.453	0.454	0.627 **	0.699 **	0.539 *
	ML	0.126	0.159	0.309	0.181	0.090	0.279	0.254	0.122	0.391
	CC	−0.247	−0.286	−0.367	−0.251	−0.190	−0.368	0.093	−0.129	−0.072
**ACSH**	AP	0.287	0.197	0.242	0.395	0.302	0.402	0.368	0.207	0.330
	ML	0.663 **	0.516 *	0.612 **	0.599 **	0.497 *	0.486 *	0.553 *	0.466 *	0.372
	CC	0.172	0.094	0.337	0.346	0.224	0.451	0.431	0.506 *	0.530 *
**iHR**	AP	0.423	0.507 *	0.605 **	0.196	0.221	0.361	0.365	0.433	0.391
	ML	0.149	0.319	0.356	−0.143	−0.127	−0.019	0.109	0.188	0.012
	CC	0.734 **	0.733 **	0.677 **	0.693 **	0.667 **	0.658 **	0.016	0.272	0.023
**SPARC**	AP	0.205	0.051	0.170	0.384	0.308	0.411	−0.056	0.011	−0.061
	ML	0.285	0.390	0.456 *	0.195	0.192	0.160	0.086	0.092	−0.056
	CC	0.390	0.512 *	0.251	0.525 *	0.601 **	0.547 *	0.217	0.211	0.114
